# Isolation and identification of aroma producing strain with esterification capacity from yellow water

**DOI:** 10.1371/journal.pone.0211356

**Published:** 2019-02-14

**Authors:** Yen-Tso Lai, Kuan-Chen Cheng, Chia-Nuan Lai, Ying-Jang Lai

**Affiliations:** 1 Institute of Biotechnology, National Taiwan University, Taipei, Taiwan; 2 Graduate Institute of Food Science Technology, National Taiwan University, Taipei, Taiwan; 3 Department of Medical Research, China Medical University Hospital, China Medical University, Taichung, Taiwan; 4 Department of Food Science, National Quemoy University, Kinmen, Taiwan; University of Oregon, UNITED STATES

## Abstract

Kaoliang is a refreshing fragranced type of Chinese spirits with slight apple fragrance that comes from ethyl acetate (EA). Special aromas are produced by esterification microorganisms, which affect the taste and quality of the wine. In this study, new yeast strains were isolated from yellow water, a by-product during fermentation process. Meanwhile, the optimal culture condition was determined for its growth and EA production. Three new strains, *Kazachstaniaexigua*, *Candida humilis* and *Saccharomyces cerevisiae* were identified from yellow water. Among these strains, *S*. *cerevisiae* S5 was the new and dominant strain. Results from response surface methodology showed that *S*. *cerevisiae* S5 produced 161.88 ppm of EA, in the medium with 4.91% yeast extract, 9.82% peptone, and 20.91% glucose after 96 hours of cultivation at 27.53°C. GC analysis showed that aroma compounds, such as EA, isoamyl acetate and 2-phenylethanol increased from the sample of optimal condition when compared to the one from initial fermentation condition.

## Introduction

Kaoliang is made from wheat-based koji, sorghum as substrate for solid-state fermentation and distillation to produce fragranced Chinese spirits [1, 2]. During fermentation, kaoliang generates various flavors such as fruits, flowers and grass aroma after blending and aging [3]. Subsequently, microorganisms conduct liquefaction, saccharification, and fermentation, resulting in the production of yellowish brown liquid, referred as yellow water, which is rich in aroma compounds and microbial flora [4]. Ethyl acetate (EA) is the major aroma compound found in kaoliang. Sensory evaluation of EA showed similarity with apple aroma that produced by microbial fermentation and metabolism [1]. Yeasts and *Aspergillus* spp. are the main microorganisms involved in brewing process [5]. *Aspergillus* produced amylase to degrade starch into smaller molecule such as carbohydrate and dextrin, yeast conducts alcohol fermentation to produce esters [6]. Ester-producing yeasts are referred as esterification microorganisms, such as *Saccharomyces rouxii*, *Hansenula anomala* and *Pichia anomala* [7, 8].

Cultivation optimization is a key element to optimize the production of bioactive components. The effects of initial pH, carbon source, nitrogen source, inoculation density and temperature have been investigated to optimized aroma and biomass production in wine making [9–12]. Compared to one-factor-at-a time approach, response surface methodology (RSM) is a statistical approach based on the fit of polynomial regression model, which can be applied to validate not only the value of independent variables but also the interaction among them [13, 14]. RSM has been applied for both evaluation of microorganism growth and metabolites production such as polysaccharides, proteins and organic compounds [15–17].

The purpose of this study is to isolate and identify new yeast strains with esterification capacity from sorghum yellow water. The optimal fermentation condition for the news strains to produce aroma compounds especially EA using RSM was evaluated.

## Materials and methods

### Isolation and purification of microorganisms

Yellow water samples used in this study was provided by the private winery in Zhongxing market (Kinmen). Sample was maintained at 4°C until use. Yeast extract peptone glucose (YEPG) agar is a selective medium used for isolation of eukaryotic microorganism [18] which is composed of 1% yeast extract, 2% peptone, 2% glucose and 2.5% agar, supplemented with 100 mg/L chloramphenicol and 50 mg/L chlortetracycline to inhibit the growth of prokaryotic microorganisms [19]. Serial dilution of yellow water samples were cultured on YEPG agar plate, at 28°C for 36 hours. Selection of strains was performed based on morphological observation. Selected strains were sub-cultured on YEPG agar by streak plate method to obtain single colony. Each strain was sub-cultured to new YEPG agar every two weeks for culture maintenance. Long-term storage of strain was done by adding 20% glycerol into the liquid culture and stored at -80°C.

### Strain identification

*S*. *cerevisiae* 21447 purchased from Bioresource Collection and Research Center (BCRC, Hsinchu city, Taiwan) was used as standard strain for physiological and biochemical characteristics. Isolated yeasts were identified by comparing DNA sequences using API 20 C AUX yeast identification kit (BioMérieux, Inc., Marcy-l'Étoile, France). The strain characteristics were done by comparing with database as described previously [20]. Strain DNA extraction was conducted as described by Doyle [21]. The 5.8S rDNA amplification was performed using Internal Transcribed Spacer (ITS), ITS1 (5’ TCC GTA GGT GAA CCT GCG G 3’) and ITS4 (5’ TCC TCC GCT TAT TGA TAT GC 3’) [22]. PCR was then performed using Phusion high-fidelity PCR master mix (New England BioLabs, Inc., Massachusetts, USA). DNA sequencing was subsequently performed and the sequences comparisons were then analyzed with Basic Local Alignment Search Tool (BLAST) software (National Center for Biotechnology Information, Maryland, USA).

### Yeast fermentation

Yeast culture was produced by inoculating single colonyto 100 ml YEPG medium at 28°C, 150 rpm for 24 hours. The YEPG medium was sterilized medium at 121°C for 20 minutes to avoid possible microbial contamination. This culture was further centrifuged at 3,824 g for 8 minutes. The supernatant was removed and yeast was adjusted to 5% (w/w) with new YEPG medium. Fermentation culture was made by inoculating 1% (v/v) yeast into YEPG medium which supplemented with 14% glucose. This culture was fermented at 28°C, 150 rpm for 96 hours.

### Response surface methodology for optimal fermentation

Determination of EA concentration of fermentation culture was conducted using three factors and three levels of Box-Behnken Design. Three factors used in RSM optimization were temperature (X_1_), nitrogen source (X_2_), glucose (X_3_). The ranges were 25–35°C, 6–24% and 10–30%, respectively. Optimal fermentation condition was determined based on EA production. Statistical analysis was conducted using Minitab (Minitab Inc., State College, PA, USA).

### Ethyl acetate (EA) extraction and analysis

Fifty ml of fermentation culture was centrifuged at 3,824 g at 4°C for 8 minutes. Twenty ml of the supernatant with 20 ppm of pentylalcohol as internal standard were mixed with 20 ml of dichloromethane for 30 seconds. The mixture was then centrifuged at 4°C for another 8 minutes. Dichloromethane layer was taken for sampling. The procedures were conducted in triplicates. The collected extract was added with sodium sulfate and filtered through filter paper. Filtrate was vacuum concentrated and stored at -20°C. Determination of EA was performed by GC/MS (GC7890/MS5975, Agilent Technologies, Santa Clara, CA, USA). The column used in this study is HP-5MS (Agilent Technologies, Santa Clara, CA, USA).

### Statistical analysis

All experiments were conducted with three independent evaluations, with three replications of each. The values were expressed as mean ± standard deviation. The RSM method followed the Box–Behnken design 3-level-3-factor with 5 center point replications. Microsoft Excel was used for the data analysis (Microsoft, Redmond, Washington, USA). Statistical Analysis System (SAS Institute Inc., Cary, North Carolina, USA) was used for T test and Duncan's new multiple range test. Minitab Statistical Software (Minitab Inc., University City, Pennsylvania, USA) was used for RSM evaluation and analysis. Statistical significant differences were all p < 0.05.

## Results

### Isolation and identification of yeasts

**Fig 1:**
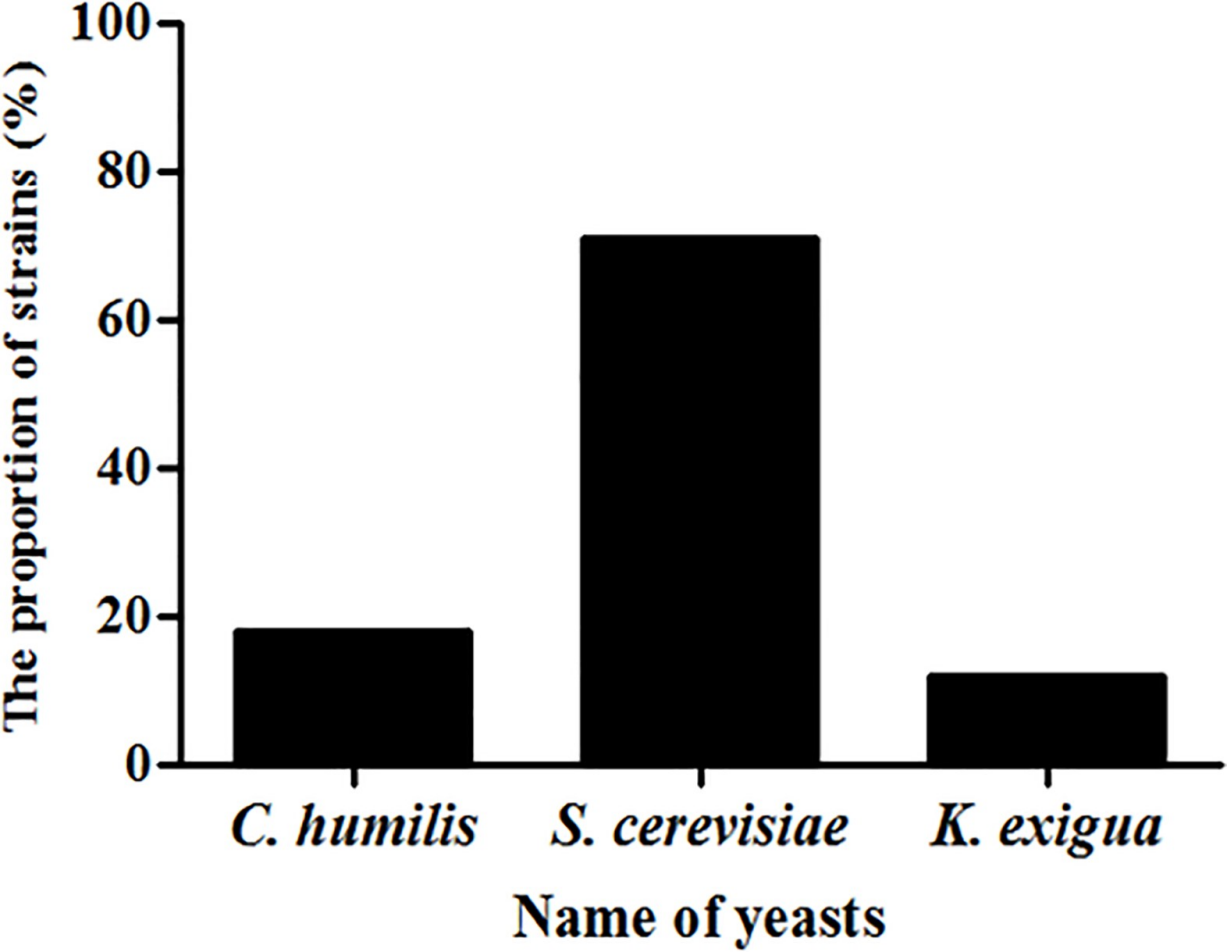
The proportion of three yeast strains isolated from yellow water.

According to the morphological characteristics, biochemical reactions and DNA sequencing, three yeast strains were isolated from the yellow water, and they were identified as *Candida humilis*, *Saccharomyces cerevisiae* and *Kazachstania exigua*. *S*. *cerevisiae* accounted for 70.59% of the total isolates, which was the dominant strain (Fig 1).

### Strain selection with ethyl acetate production ability

**Fig 2:**
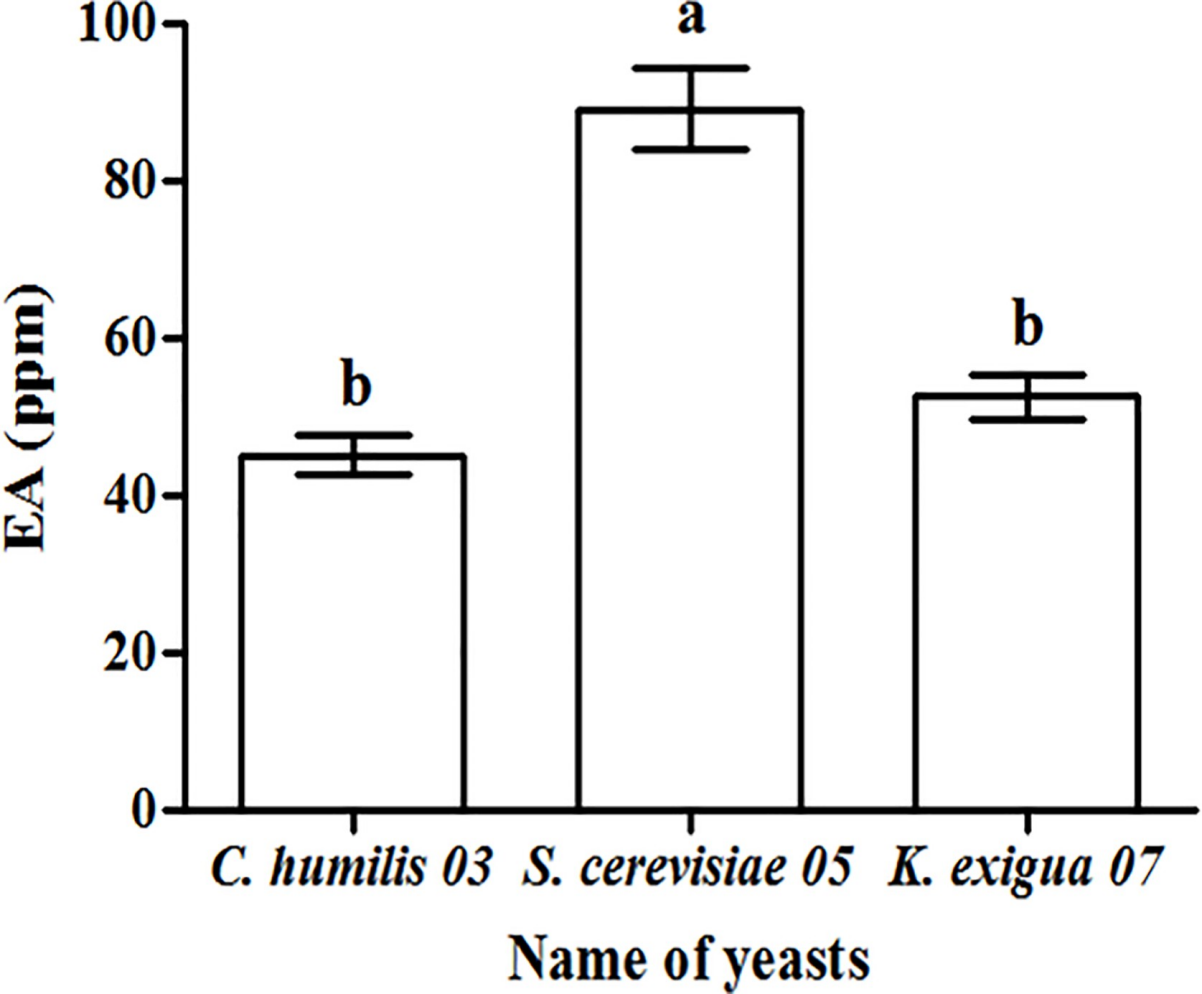
The EA concentrations of fermented broths from three yeast strains.

The fermented broths of three isolated strains, *C*. *humilis* 03, *S*. *cerevisiae* 05 and *K*. *exigua* 07 were analyzed for EA contentand the results were summarized in Fig 2. The EA content from *C*. *humilis* 03, *S*. *cerevisiae* 05 and *K*. *exigua* 07 samples were 45.14, 89.08 and 52.51 ppm, respectively. All strains exhibited the esterification ability, and *S*. *cerevisiae* 05 was the highest of them.

*S*. *cerevisiae* 05 was chosen for the subsequent study for the following reasons: (1) Safety: *S*. *cerevisiae* is a safe and stable food microorganism belonging to general recognized as safe (GRAS) strains as Food and Drug Administration (FDA) announced, which has long been used for baking and brewing [23]; (2) dominant species: *S*. *cerevisiae* accounted for 70.59% in yellow water microorganism population, indicating that the fermentation environment was suitable for *S*. *cerevisiae*, which may even had the ability to inhibit the growth of pathogens.

### Physiological and biochemical characteristics of *S*. *cerevisiae*

Result of carbon source assimilation showed *S*. *cerevisiae* 05 and *S*. *cerevisiae* 21447 had no significant difference in carbon source preference (Table 1), and both of them were identified as *S*. *cerevisiae* in 99.9% probability (S1 Fig).

**Table 1:** The result of carbon source assimilation in yeast identification system API 20 C AUX tests.

Tests	Active ingredients	S5	BCRC#21447
0	none	**-**	**-**
GLU	D-glucose	**+**	**+**
GLY	glycerol	**-**	**-**
2KG	calcium 2-keto-gluconate	**-**	**-**
ARA	L-arabinose	**-**	**-**
XYL	D-xylose	**-**	**-**
ADO	adonitol	**-**	**-**
XLT	xylitol	**-**	**-**
GAL	D-galactose	**+**	**+**
INO	inositol	**-**	**-**
SOR	D-sorbitol	**-**	**-**
MDG	methyl-αD-glucopyranoside	**+**	**+**
NAG	N-acetyl-glucosamine	**-**	**-**
CEL	D-cellobiose	**-**	**-**
LAC	D-lactose	**-**	**-**
MAL	D-maltose	**+**	**+**
SAC	D-saccharose	**+**	**+**
TRE	D-trehalose	**+**	**+**
MLZ	D-melezitose	**+**	**+**
RAF	D-raffinose	**+**	**+**
H/PH^+^	produced mycelium	**-**	**-**

(-) means negative reaction, and (+) means positive reaction.

**Fig 3:**
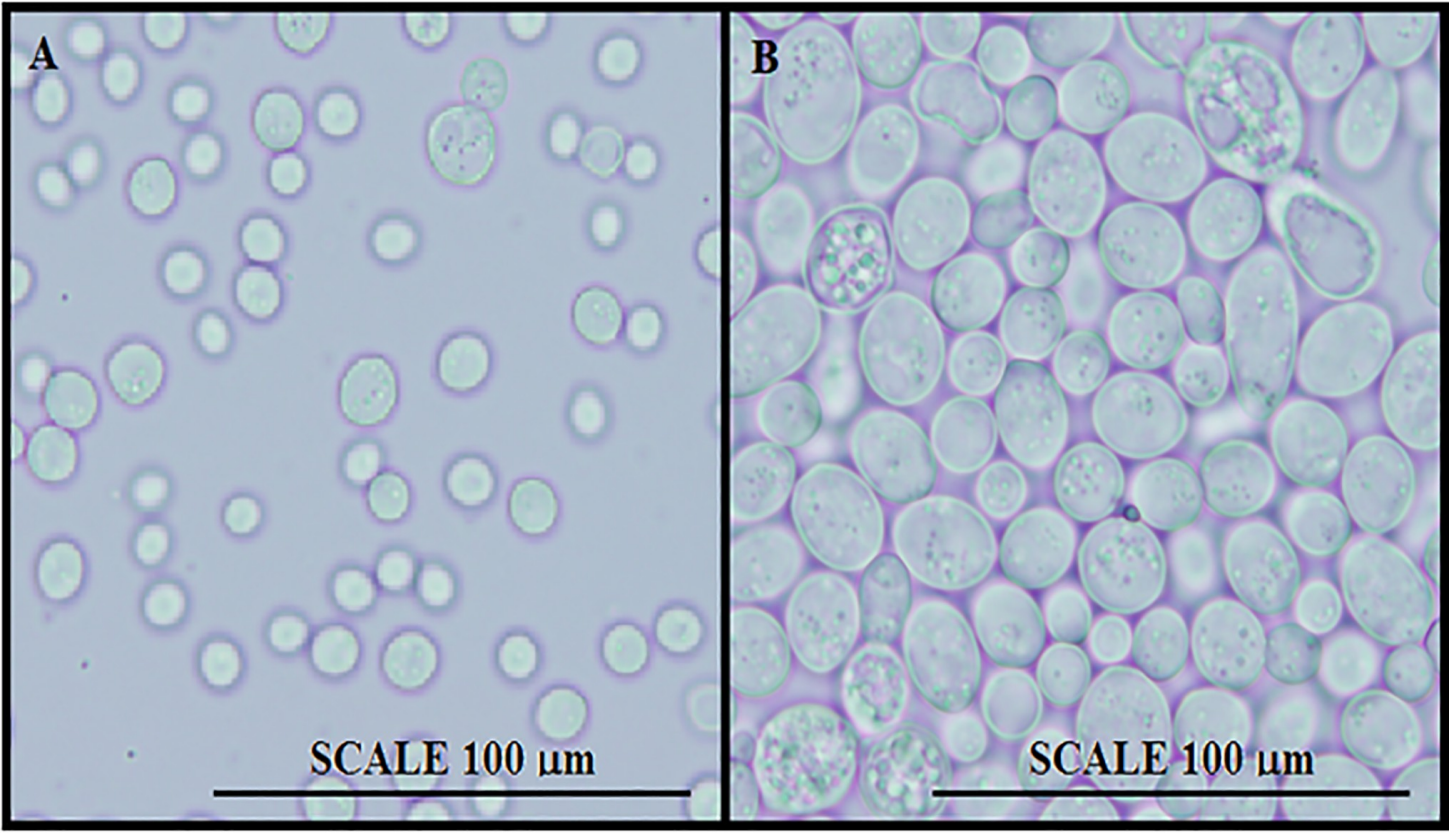
The appearance of S5 (A) and BCRC21447 (B) under microscopy.

Experiments such as microscope inspection, carbon and nitrogen assimilation, carbohydrate fermentation, high glucose tolerance and cycloheximide resistance were carried out and the results were summarized in Table 2. The only difference was the appearance of the strains as shown in Fig 3. It is evident that the size of S5 strain was smaller than that of the BCRC 21447 strain. The diameters of S5 and BCRC 21447 strains were approximately for 6.3 and 10.0 μm. This phenomenon explained the rapid growth of the S5 strain, where the generation time of the S5 and BCRC 21447 strains were 73.2 and 93.7 minutes, respectively.

**Table 2:** The result of “Yeasts: Characteristics and identification tests”.

Appearance of yeasts	Paper result	S5	BCRC#21447
Mycelium	-	-	-
Ascospore	+	+	+
Ballistospore	-	-	-
Carbon source assimilation tests	Paper result	S5	BCRC#21447
D-galactose	**+**	**+**	**+**
D-glucosamine	**-**	**-**	**-**
D-xylose	**-**	**-**	**-**
L-arabinose	**-**	**-**	**-**
D-saccharose	**+**	**+**	**+**
D-maltose	**+**	**+**	**+**
D-cellobiose	**-**	**-**	**-**
Salicin	**-**	**-**	**-**
D-lactose	**-**	**-**	**-**
starch	**-**	**-**	**-**
glycerol	**-**	**-**	**-**
D-mannitol	**+**	**+**	**+**
Lactic acid	**-**	**-**	**-**
succinic acid	**-**	**-**	**-**
citric acid	**-**	**-**	**-**
methanol	**-**	**-**	**-**
ethanol	**+**	**+**	**+**
L-fructose	**+**	**+**	**+**
D-raffinose	**+**	**+**	**+**
Nitrogen source assimilation tests	Paper result	S5	BCRC#21447
Sodium nitrate	**-**	**-**	**-**
L-lysine	**-**	**-**	**-**
Ammonium sulfate	**+**	**+**	**+**
Sugar fermentation tests	Paper result	S5	BCRC#21447
D-glucose	**+**	**+**	**+**
D-galactose	**+**	**+**	**+**
D-maltose	**+**	**+**	**+**
D-saccharose	**+**	**+**	**+**
D-lactose	**-**	**-**	**-**
D-cellobiose	**-**	**-**	**-**
starch	**-**	**-**	**-**
L-fructose	**+**	**+**	**+**
D-raffinose	**+**	**+**	**+**
Glucose solution osmolaritytests	Paper result	S5	BCRC#21447
40% glucose solution	**+**	**+**	**+**
50% glucose solution	**+**	**+**	**+**
60% glucose solution	**-**	**-**	**-**
cycloheximide test	Paper result	S5	BCRC#21447
100 ppm	**-**	**-**	**-**
1000 ppm	**-**	**-**	**-**

### Response model fitting and adequacy checking

The medium composition and the fermented parameters certainly affect the EA yield of yeasts [24]. Different yeasts prefer specific cultivation system. In this study, three factors namely fermented temperature (X_1_), nitrogen sources (X_2_), and glucose (X_3_) on EA production were investigated using BBD-RSM design (Table 3). The nitrogen sources were yeast extract and peptone, and the ratio of them was kept at 1:2. The results of BBD were summarized in Table 4 and analyzed by multiple regression analysis. Following second-order polynomial equation was obtained.

$$Y_{EA}=153.84-32.49x_{1}-2.53x_{2}+0.17x_{3}-33.77{x_{1}}^{2}-31.20{x_{2}}^{2}-24.14{x_{3}}^{2}+0.15x_{1}x_{2}-8.40x_{1}x_{3}+2.12x_{2}x_{3}$$ (Eq 1)

**Table 3:** Levels of factors chosen for the Box-Behnken design.

Factors	Symbols	Coded levels		
		-1	0	+1
Temperature (^o^C)	X_1_	25	30	35
Nitrogen sources (%)*	X_2_	6	15	24
Glucose (%)	X_3_	10	20	30

* The nitrogen source was composed by peptone and yeast extract. (Peptone: Yeast extract = 2:1)

**Table 4:** Box-Behnken design matrix and experimental results of EA production and the theoretical EA production of equation.

Run Order	Factors			EA(ppm)	Theoretical EA
	X_1_	X_2_	X_3_		
1	35	15	30	63.00	55.10
2	25	6	20	127.15	123.79
3	30	15	20	150.03	153.53
4	35	15	10	72.64	71.52
5	25	24	20	124.34	118.44
6	30	24	30	93.60	98.07
7	30	15	20	152.15	153.53
8	30	15	20	161.47	153.53
9	25	15	10	112.06	119.60
10	35	24	20	50.89	53.89
11	30	6	30	96.94	98.88
12	30	15	20	153.72	153.53
13	30	15	20	151.83	153.53
14	30	6	10	107.66	102.77
15	30	24	10	95.82	93.49
16	25	15	30	136.01	136.71
17	35	6	20	53.11	58.64

X_1_ = Temperature; X_2_ = Nitrogen source; X_3_ = Glucose

The analysis of variance for the model is shown in Table 5, and the fitness of it was examined using the determination coefficient (R^2^ = 0.964), which suggests that the sample variation of 96.4% for EA production was associated with the variable factors. In addition, the lack of fit for the model was insignificant (*p*> 0.05), verifying the accuracy fit of the second-order model (Eq 1) to the true response of EA production. Moreover, the *F* value of 49.19 and *p* value < 0.05 for the regression, supporting the second-order model, adequately approximated the response surface. As a result, canonical analysis demonstrated that the predicted maximum of EA production was 161.88 ppm at fermented temperature 27.5°C, 14.73% nitrogen sources, and 20.91% glucose. The results clearly indicated that all these variables influenced EA yield.

**Table 5:** Analysis of variance (ANOVA) for response surface quadratic model.

Source	Coefficient	Degree of freedom	Sum of squares	*F* value	*P* value
Constant	153.84				
*X*_*1*_	-32.49	1			0.000
*X*_*2*_	-2.53	1			0.339
*X*_*3*_	0.17	1			0.946
*X*_*1*_^2^	-33.77	1			0.000
*X*_*2*_^2^	-31.20	1			0.000
*X*_*3*_^2^	-24.14	1			0.000
*X*_*1*_*X*_*2*_	0.15	1			0.967
*X*_*1*_*X*_*3*_	-8.40	1			0.047
*X*_*2*_*X*_*3*_	2.12	1			0.561
Regression		9	21,449.5	49.19	0.000
Linear		3	8,495.9	58.45	0.000
Square		3	12,653.3	87.05	0.000
Interaction		3	300.2	2.07	0.193
Residual		7	339.2		
Lack-of-Fit		3	259.5	4.34	0.095
Pure Error		4	79.7		
Total		16	21,788.6		

### Effects of factors on EA production

**Fig 4:**
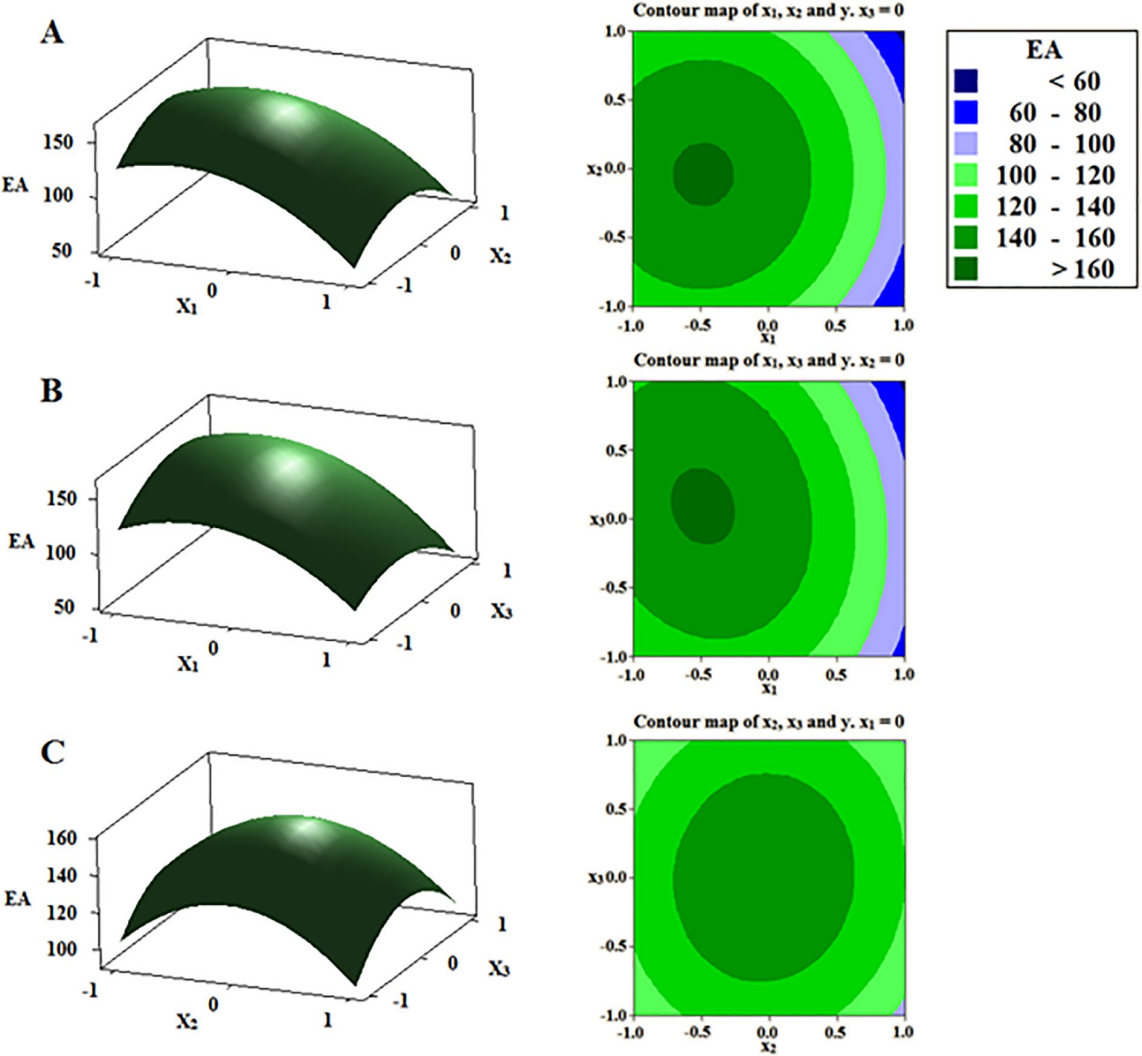
Three-dimensional surface and contour plots of three factors on EA production. The effect of temperature (X_1_) and nitrogen sources (X_2_) (A), temperature (X_1_) and glucose (X_3_) (B), and nitrogen sources (X_2_) and glucose (X_3_) (C) on EA production.

According to the mathematical model, three-dimensional surface and contour plots were generated to reveal the interaction among the three independent variables studied and to depict the combined effects of these variables on EA production. As the influence of two variables on response surface was plotted, the other variable was kept at its zero level. As shown in Fig 4, EA production gradually increased with the increase of fermented temperature, nitrogen sources and glucose. However, it was found that the yield of EA will decrease while these three factors continuously increased (Fig 4). These results demonstrated that our response surface generated from a quadratic model was defined as maximum surface.

### Verification of optimization

To validate the predicted *S*. *cerevisiae* S5 EA production, verification fermentation with the predicted optimal value of variables was carried out.As shown in Table 6, a high correlation between predicted (161.88 ppm) and experimental yield of EA was observed. The EA production was 163.22 ppm after the 96-hours fermentation, which was approximately 1.83 times higher than the one from basic medium (Table 6).

**Table 6:** Comparison of basic and optimum medium on EA production.

Factors	Basic medium	Optimum medium
Temperature (^o^C)	28.0	27.5
Nitrogen source (%)	3.00	14.73
Glucose (%)	14.00	20.91
EA yield (ppm)^a^	89.08 ± 9.09	163.22 ± 19.15

EA = ethyl acetate.

^a^The results are presented as the means of five replicated.

### Content of aroma compounds in fermented broth

We compared the aroma compounds between the fermented broths from basic and optimum medium (Table 7). Results showed that the content of major aroma compounds, such as EA, acetoin, isoamyl alcohol, isoamyl acetate, 2-methylbutyl acetate, 2-phenylethanol, 2-phenethyl acetate and tryptophol, increased after optimization, and the increase ratio were between 1.28–2.01.

**Table 7:** Comparison of basic and optimum fermented broth content of aroma compounds (ppm).

Aroma compounds	Basic medium	Optimum medium	Increase fold
Ethyl acetate	90.29 ± 9.32	168.43 ± 16.64	1.87
Acetoin	116.47 ± 4.22	219.92 ± 12.95	1.89
Isoamyl alcohol	113.17 ± 11.24	159.25 ± 13.84	1.41
Isoamyl acetate	58.98 ± 2.57	118.84 ± 4.36	2.01
2-Methylbutyl acetate	N/A	15.15 ± 1.67	-
2-Phenylethanol	401.86 ± 35.40	515.42 ± 54.12	1.28
2-Phenethyl acetate	29.91 ± 1.09	48.17 ± 3.04	1.61
Tryptophol	53.09 ± 4.39	68.88 ± 5.69	1.29

EA = ethyl acetate.

The results are presented as the means of three replicated.

## Discussion

*S*. *cerevisiae* also known as brewing yeast and baking yeast is profoundly used in food industry [25]. Since the whole genome sequence of *S*. *cerevisiae* has been elucidated, it has been used as model for disease prevention and molecule biology [26]; more than that, *S*. *cerevisiae* can produce many organic compounds such as ethanol, lactic acid, glycerol and EA [27]. *C*. *humilis* was often used for fermenting traditional Italian rye sour dough with *Lactobacillus* spp. [28], both *C*. *humilis* and *L*. spp. exhibits acid-fastness and salt-tolerance properties. *C*. *humilis* can inhibit growth of other bacteria and eventually became the dominant species during fermentation [29]. *C*. *humilis* can also produce aroma compounds such as ethanol, acetaldehyde and EA [30]. *K*. spp. are common in pineapple, star fruits etc. [31], which can be used for making Turkish kefir [32]. *K*. spp. can also be used as feed additive which will reduce the poultry infection of Salmonella [33]. Etienne-Mesmin et al. [34] found a new probiotic yeast strain, *S*. *cerevisiae* CNCM I-3856, which can inhibit the growth of *Escherichia coli* O157:H7 in digestive system. Fadel et al. [35] isolated a new thermotolerant strain *S*. *cerevisiae* F-514 from Egyptian distillery factory, which could improve ethanol yield by fermenting sugarcane molasses. Parapouli et al. [36] successful induced several new enzymes from new yeast strain *S*. *cerevisiae* Z622 which could contribute to a better understanding of how *S*. *cerevisiae* cells adapt to wine fermentation.

Esterification involves many enzyme reactions such as alcohol acetyltransferase (AAT) [37] and is also regulated by many genes such as alcohol acetyltransferase gene (*ATF1*) [38]. EA is the major aroma compound in kaoliang [39], and it’s also a crucial esterification component of yeasts [40]. Yeasts with higher EA production efficiency means that the higher amount of ester aroma compounds were produced during the winemaking process.

Fermentation time is an important parameter in fermentation engineering since the microorganisms affect the composition of the fermentation broth. In this study, we performed an eight-day-fermentation and monitored EA production every 24 hours. The results showed that EA production reached the highest on the fourth day and then declined (S2(A) Fig). Glucose also played an important role on EA synthesis. In alcoholic fermentation, glucose could be converted to pyruvate by glycolysis pathway, and produce ethanol in an anaerobic environment [41]. In this process, glucose not only provides the energy for growth, but also produces the ethanol for EA synthesis. There were three metabolic pathways to synthesize EA: esterification [40], hemiacetal reaction [42] and alcoholysis reaction [43], and these metabolic pathways require ethanol to participate the action. However, excess glucose had an inhibitory effect in the yield of EA that might be attributable to unfavorable osmotic pressure (S2(B) Fig). The sluggish growth of microorganisms was often observed at high osmotic pressures, while initial sugar concentration exceeded a certain level [41].

Organic nitrogen source was widely used in the cultivation of yeasts, suggesting that essential amino acid could be synthesized from organic nitrogen sources instead of inorganic ones [44]. The biochemical characteristics results of this study also supported that an organic nitrogen source (yeast extract and peptone) was more favorable for biomass production in *S*. *cerevisiae* S5 than an inorganic nitrogen source (ammonium sulfate). Yeast extract and peptone are rich in vitamins, essential amino acids and trace elements such as magnesium cation. Magnesium cation acts as a mainly co-factor of several enzymes of fermentation metabolism and protecting yeast cells from stressful conditions [45]. The yield of EA increased with the elevation of nitrogen source, but excess nitrogen source may also suppress EA production (S2(C) Fig). It was supposed that high concentrations of cations would decrease the amount of live cells during the fermentation process, which results the decrease in alcoholic content and fermentative efficiency [46]. In addition, certain by-products existing at high concentrations of yeast extract and peptone would lead to abatement in EA yield, such as L-pyroglutamate [47].

Fermented temperature was associated with the yield of EA (S2(D) Fig). Previous study suggested that, in fermentation process, temperature would influence the yeasts’ gene regulation [48], target product yield [49] and enzyme activity [50]. Each yeast strain had distinct cultivation temperature. It was generally believed that the EA-synthesis enzymes had higher activity at 30°C [51], which was in agreement with our results.

Many studies discussed about winemaking concerning *S*. *cerevisiae* and the concentration of EA. Mateos et al. [52] used nine *S*. *cerevisiae* strains in winemaking and found that the concentration of EA in wine was 44.1–56.9 ppm. Roza et al. [53] explored the biomass, sugars and ethanol influence the aroma during the cider industrial fermentation, and found that the concentration of EA was about 44 ppm.The previous study indicated that the concentration of EA in wines is generally lower than 150 ppm. When EA went above 200 ppm will be considered negative for the wine aroma [54]. Therefore, *S*. *cerevisiae* S5 was regarded as a winemaking strain with acceptable EA production.

The EA assists *S*. *cerevisiae* to disseminate to the environment by attracting insects [55]. These aroma compounds also play important roles in wine. In winemaking, for example, carbonic maceration process could induce cell wall hydrolysis, which generated esters, such as isoamyl acetate [56], which is one of the esters remarkably contributes to the aroma profile of white wines [57]. Therefore, medium optimization provides a reinforced approach to produce aroma compounds.

## Conclusion

The current study screened a new yeast strain, *S*. *cerevisiae* S5, with winemaking-potentiality from Kinmen kaoliang yellow water. Furthermore, the optimum value of independent variables (fermented temperature, nitrogen sources and glucose) for EA production was evaluated and predicted by BBD-RSM. As a result, fermentation conditions of 27.5°C, 14.73%, and 20.91% were suggested for EA production. EA production was 163.22 ppm at RSM-optimized medium, which was 100.83% of the software-predicted value. It is noteworthy that the content of many aroma compounds were increased in the optimum medium compared with those obtained from basic medium.

In conclusion, this study reported a new yeast strain with enhanced aroma production ability, which provides a new insight into the aroma compounds production or winemaking industrial application of *S*. *cerevisiae*.

## Supporting information

S1 Fig The results of yeast identification system API 20 C AUX test. Both *S*. *cerevisiae* 05 and *S*. *cerevisiae* 21447 were identified as *S*. *cerevisiae* in 99.9% probability.
(TIF)

S2 Fig The correlation of fermentation time and EA concentration. The highest EA concentration at 100.75 ppm in fermented broth was obtained on the 4^th^ day (A).The correlation of carbon source concentration and EA concentration. The treatment of glucose 20% yielded the highest EA concentration 120.20 ppm in fermented broth (B). The correlation of nitrogen source concentration and EA concentration. The treatment nitrogen 12% yielded the highest EA concentration 125.96 ppm in fermented broth (C). The correlation of fermented temperature and EA concentration. The temperature 30°C yielded the highest EA concentration 90.80 ppm in fermented broth (D).
(TIF)
