# Supplementary figures and images for: Isolation and identification of aroma producing strain with esterification capacity from yellow water
Source: PLoS One. 2019 Feb 14;14(2):e0211356. doi: 10.1371/journal.pone.0211356 (PMC6375555; doi:10.1371/journal.pone.0211356)

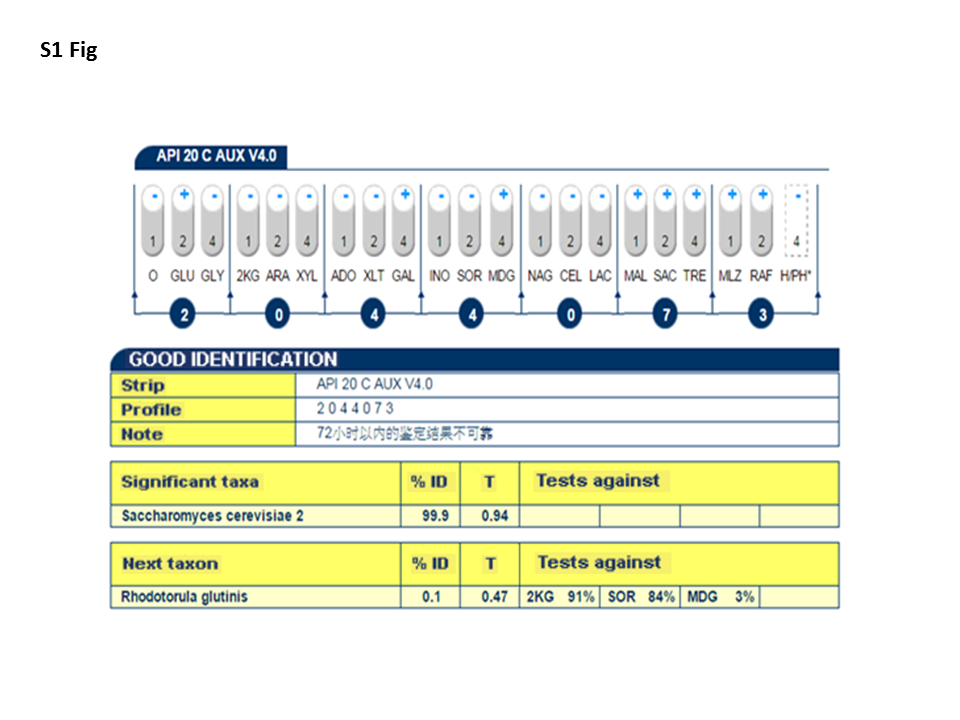

Supplement: S1 Fig — Both S. cerevisiae 05 and S. cerevisiae 21447 were identified as S. cerevisiae in 99.9% probability. (TIF) [file pone.0211356.s001.TIF]

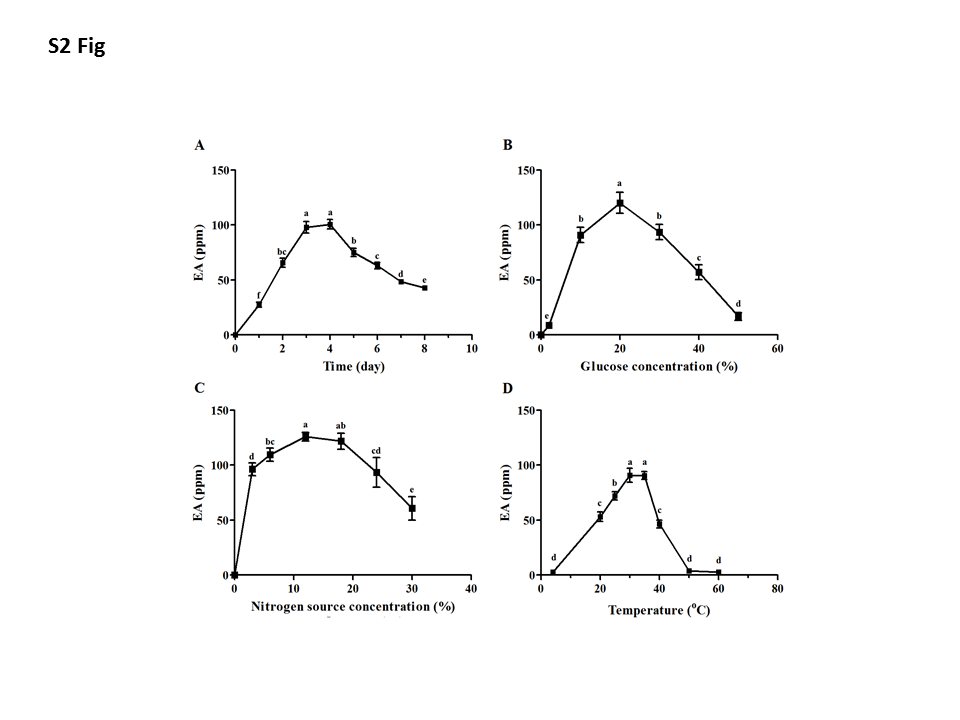

Supplement: S2 Fig — The highest EA concentration at 100.75 ppm in fermented broth was obtained on the 4th day (A).The correlation of carbon source concentration and EA concentration. The treatment of glucose 20% yielded the highest EA concentration 120.20 ppm in fermented broth (B). The correlation of nitrogen source concentration and EA concentration. The treatment nitrogen 12% yielded the highest EA concentration 125.96 ppm in fermented broth (C). The correlation of fermented temperature and EA concentration. The temperature 30°C yielded the highest EA concentration 90.80 ppm in fermented broth (D). (TIF) [file pone.0211356.s002.TIF]
